# Introduction of a digital near-vision reading test for normal and low vision adults: development and validation

**DOI:** 10.1186/s40662-020-00216-0

**Published:** 2020-10-22

**Authors:** Georgios Labiris, Eirini-Kanella Panagiotopoulou, Eleftherios Chatzimichael, Maria Tzinava, Asimina Mataftsi, Konstantinos Delibasis

**Affiliations:** 1 Department of Ophthalmology, University Hospital of Alexandroupolis, Dragana, 68100 Alexandroupolis, Greece; 2 Department of Computer Science and Biomedical Informatics, University of Thessaly, 35100, Lamia, Greece; 3 2nd Department of Ophthalmology, Aristotle University of Thessaloniki, 56429, Thessaloniki, Greece

**Keywords:** Digital near-vision chart, Critical print size, Reading acuity, Reading speed, Presbyopia, Low vision

## Abstract

**Background:**

MNREAD is an advanced near-vision acuity chart that has already been translated and validated in Greek language. Considering that no validated Greek digital near-vision test exists, our primary objective was to develop and validate a digital near-vision reading test based on the fundamental properties of the Greek printed MNREAD (MNREAD-GR).

**Methods:**

This is a prospective, comparative study. A digital near-vision chart was developed (Democritus Digital Acuity Reading Test – DDART) with text size calibration, audio recording for automatic reading timing, as well as automatic calculation of reading acuity (RA), maximum reading speed (MRS), critical print size (CPS) and reading accessibility index (ACC). Normal and low vision subjects participated in the validation process, responding to MNREAD-GR and DDART at the same day, at a 40 cm viewing distance. Differences in all parameters between the charts were compared with t-test and intraclass correlation coefficients (ICCs). Within 15 days, all participants responded again to DDART in a different set of sentences to assess its test-retest reliability.

**Results:**

One hundred patients (normal vision group - NVG: 70 patients; low vision group - LVG: 30 patients) responded to both reading tests. Non-significant differences were detected for all parameters between DDART and MNREAD-GR except for MRS and ACC that were significantly higher in MNREAD-GR in NVG (*p* <  0.01). NVG participants demonstrated sufficient ICCs that ranged from 0.854 to 0.963, while LVG demonstrated ICCs for RA, ACC, MRS and CPS equal to 0.986, 0.894, 0.794 and 0.723, respectively. All parameters calculated with DDART demonstrated excellent test-retest reliability (ICCs: 0.903 – 0.956).

**Conclusions:**

The proposed reading test presented comparable validity and repeatability to MNREAD-GR suggesting that it can be used both in normal and low vision Greek patients.

**Trial registration:**

ClinicalTrials.gov, NCT04242836. Registered 24 January 2020 – Retrospectively registered.

## Background

Reading is a fundamental activity of daily living that reflects the overall vision capacity [1]. Therefore, it is no surprise that the evaluation of the reading ability is among the routine tests in a standard ophthalmological examination. However, some reading tools that we use in clinical settings (i.e. the Jaeger charts) fail to reflect the whole spectrum of reading capacity and provide information only on the near vision discriminant ability [2].

To address the aforementioned discrepancy, advanced reading tools like the MNREAD or the RADNER Reading Charts have been developed and provide a more comprehensive way to estimate near-vision capacity with the use of a series of novel parameters [3–10]. MNREAD, which was developed by the Minnesota Low Vision Laboratory, evaluates reading ability by four distinct but inter-related parameters: a) reading acuity (RA), defined as the smallest print that can be read, b) critical print size (CPS) that is the smallest print that can be read in maximal speed, c) maximum reading speed (MRS), and d) reading accessibility index (ACC) that represents the patient’s access to commonly encountered printed material [11]. The MNREAD uses sentences with the same number of characters (60 including spaces), however with slightly varying total number of words and different length of individual words, presenting random characteristics in terms of lexical difficulty and complexity [3]. On the other hand, the RADNER Reading Chart, which also calculates reading speed for each sentence, RA, MRS and CPS, consists of standardized, highly comparable sentences regarding length and position of words, number of syllables, lexical difficulty and syntactical complexity [7].

Migration to digital reading is a reality for the Western citizens of the twenty-first century. Reading on computers, laptops and other video screens permit the easy adjustment of the text size, contrast polarity, color and font size, enabling even low or very-low vision patients to read. Moreover, video screen technology allow for the development of contemporary reading tools and applications in a single device that can be upgraded if or when the new version becomes available. However, the performance of any digital reading test depends heavily on the screen size, its display technology, and its resolution [12].

Digital versions of both the RADNER and MNREAD reading tests have been developed [13–17]. Specifically, the MNREAD has migrated to the iPad with minimal differences in the evaluated parameters [16–18]. According to the iPad, MNREAD application user guide [19], the MNREAD app supports the following features: a) display of text at logMAR +1.2 to −0.1, which requires a 264 ppi screen, b) automatic estimation of the reading parameters, with manual correction if necessary, c) varying viewing distance for low vision and normal vision. However, the MNREAD app does not provide automatic reading time measurement, by analyzing the acquired voice signal, or text size calibration that would allow the application to be used in different screens. Despite the small screen size of most of the iPad tablets, the authors suggested that the iPad MNREAD provides accurate measurements even in low vision individuals.

Nowadays, the digitalization of reading tests benefits from the automatic calculation of reading times. One of the first attempts to measure reading duration for the assessment of visual acuity was reported by Radner et al. [7, 13]. Xu et al. [20] reported on two non-automatic methods for calculating the reading time of each sentence: using an (online) stopwatch and using a cursor on the visualized waveform of the recorded patient’s audio. Dexl et al. [21] proposed a semi-automatic method to calculate reading duration (Salzburg Reading Desk) which records the patient’s voice, while the operator sets the beginning and ending time of the patient’s read-out. Calabrèse et al. [16] reported on the use of a simple automatic timer implemented as a feature of an iPad digital reading test that starts as soon as each chart is presented to the patient. Finally, Radner et al. [14] proposed an automated method of measuring the speech duration by determining the onset and the end of vocalization, while offering the capability of manually editing the results.

A Greek printed version of the MNREAD chart has been developed and validated by the Aristotle University of Thessaloniki [22]. According to the validation study, special attention was given to comply with the original chart mandates, like the crowding of sentences, and the phrases selected that should resemble normal everyday reading. All three sub-versions of the Greek MNREAD that were developed proved to be valid for comparative studies in research and clinical settings for low and normal vision patients.

Within this context, the primary objective of our study was to develop and validate a computer-based digital near-vision reading test based on the fundamental properties of the Greek version of the printed MNREAD, implementing advanced features for text size calibration and automatic timing, which make the test accurate, more efficient, and available regardless of the computer manufacturer.

## Methods

### Setting

This is a prospective, comparative trial. Study protocol adhered to the tenets of the Declaration of Helsinki and written informed consent was provided by all participants. The institutional review board of Democritus University of Thrace approved the study protocol (ID: ES3/Th2/27-03-2019). The study was conducted at the Department of Ophthalmology in the University Hospital of Alexandroupolis, Greece, between March 2019 and November 2019. Official registration number of the study is NCT04242836.

### Participants

Participants were enrolled from the outpatient service of the hospital in a consecutive-if-eligible basis. Eligibility criteria included age between 18 and 75 years with adequate literacy of written Greek language, while, exclusion criteria included dyslexia, attention-deficiency, and former diagnosis of mental and/or psychiatric diseases.

### The Greek version of the MNREAD acuity chart

The Greek version of the MNREAD acuity chart (MNREAD-GR) was developed and validated by Mataftsi and co-workers [22]. It evaluates near vision capacity with four distinct tests: a) RA, b) MRS, c) CPS, d) ACC. For methodological details of the MNREAD-GR, please refer to the corresponding publication [22]. Three versions of the MNREAD-GR have been developed with different sentences in each version. Each version consists of 19 logarithmically decreasing sentences between 1.3 logMAR and −0.5 logMAR in 0.1 logMAR steps; therefore the size-ratio between adjacent sentences remains constant. All three versions demonstrate non-significant differences in estimating the diagnostic parameters [22], therefore they are considered interchangeable and suitable for comparative studies [23]. For each sentence, the reading speed (measured in words per minute - wpm) is calculated by the following formula [22, 24, 25]:

1 $$ \mathrm{Reading}\ \mathrm{speed}=60\times \left(10- errors\right)/\left( time\kern0.5em \mathrm{in}\ \mathrm{seconds}\right), $$

where *errors* is the number of mistakes made by the patient in the current sentence and *time* (in seconds) is the patient’s reading duration of the current sentence, calculated as described in subsection 4.2. After the end of the test, four diagnostic parameters, designed to reflect the actual reading capacity of the individual, are calculated as follows [7, 22, 24–26]:

Reading acuity (RA): is defined as the smallest print that can be read by the patient easily (measured in logMAR). It is calculated by the following formula:

2 $$ \mathrm{RA}=1.4-\left(\mathrm{sentences}\times 0.1\right)+\left(\mathrm{errors}\times 0.01\right) $$

Maximum reading speed (MRS): is defined as the patient’s reading speed (measured in wpm) when reading is not limited by print size. It is calculated by averaging the reading speed of the sentences with print size larger than the CPS.

Critical print size (CPS): is defined as the smallest print size (measured in logMAR) that can be read with the MRS, i.e., with speed greater than or equal to the average reading speed of the larger logMAR print sentences minus 1.96 times the standard deviation (SD) of the reading speed of these sentences.

Reading accessibility index (ACC): is defined as the mean reading speed of the 10 largest print sizes of the MNREAD Acuity Chart at 40 cm (1.3 to 0.4 logMAR), divided by 200 wpm, which is the mean reading speed of normally sighted young adults aged 18 to 39 years old. This parameter was designed for better evaluation of one’s access to text across the range of the 10 most common print sizes found in everyday life. For instance, a value of 0 means no access to commonly encountered printed material, while 1.0 is the mean value for normally sighted young adults that indicates reading fluency within the everyday life print sizes.

### The Democritus Digital Acuity Reading Test

The Democritus Digital Acuity Reading Test (DDART) is based on the fundamental principles of the MNREAD-GR [22], however, it includes a broader set of sentences. For the validation process, the exact same set of sentences of the MNREAD-GR were used to ensure that no character/sentences-related bias could interfere with the validation process [27].

**Fig. 1:**
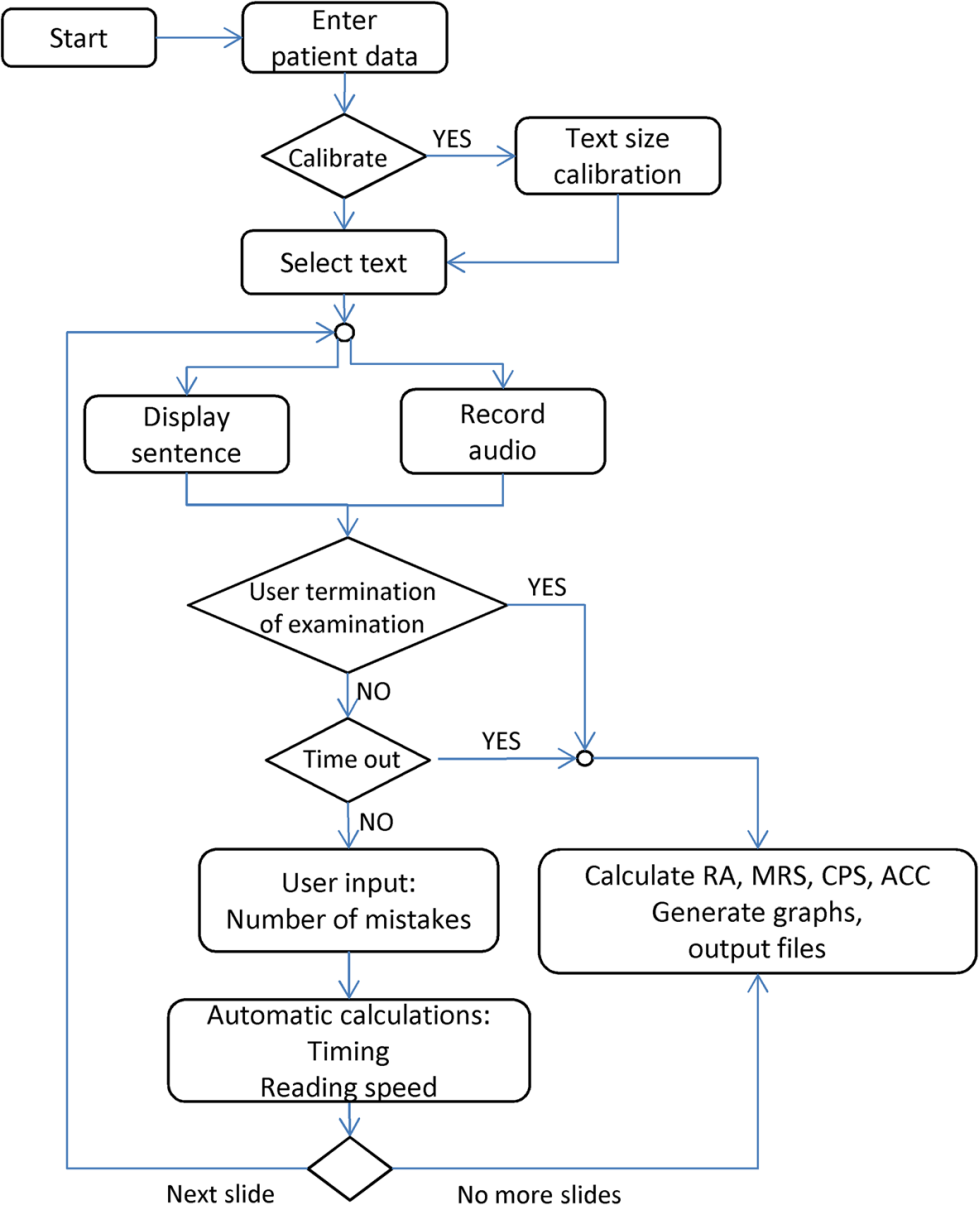
The Democritus Digital Acuity Reading Test (DDART) testing sequence

In brief, the inherent characteristics of DDART allow: a) the precise display of the reading sentences from 1.3 up to − 0.1 logMAR with step of 0.1, b) the audio recording as well as the automatic timing of the patient’s readings and the determination of reading speed for each sentence, and, c) the real-time calculation of RA, MRS, CPS, and ACC (Fig. 1).

**Fig. 2:**
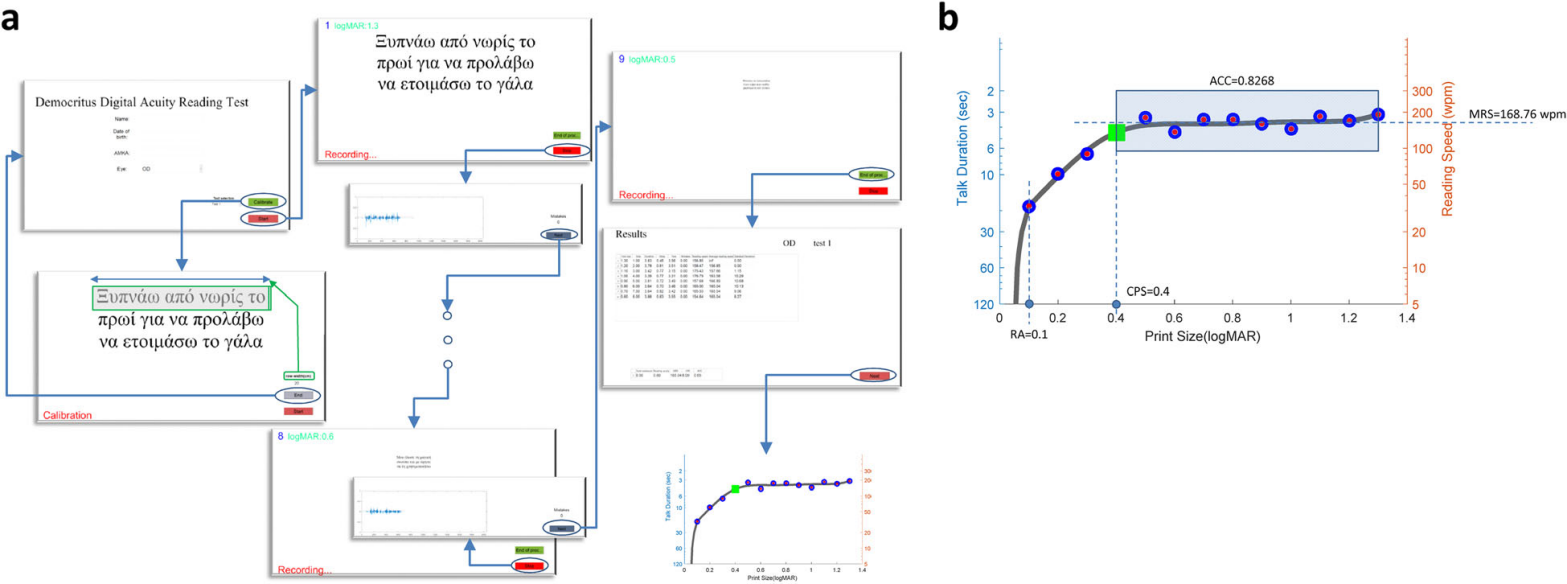
Overview of DDART software implementation. **a** A schematic workflow of the DDART, **b** the DDART curve showing the four parameters

For the development of the DDART, the MATLAB v9.0.0.341360 (2016a) programming environment (MathWorks Inc., Natick, Massachusetts) was used, which resulted in an executable program for Microsoft Windows. The actual screens at each step of the examination are shown in Fig. 2a. The initial screen for patient data input and the optional calibration screen are shown at the left part of the figure. A few essential patient data are currently supported by the proposed implementation of the digital reading test: the patient’s name, year of birth, the social identification number, and the eye indication (‘OD’, ‘OS’ or ‘OU’), as well as the set of sentences used for this specific patient. The sentences for 1.3 logMAR, up to 0.6 logMAR are cascaded in the middle part of the figure. As soon as a sentence appears, the audio recording commences until the “STOP” button is pressed. Subsequently, the recorded sound signal is displayed and the number of errors made by the patient is entered. At the rightmost part of the figure, the termination of the examination is demonstrated (in this example at 0.5 logMAR), due to patient’s inability to read the sentence (upper part). The results for each sentence (without including the sentence that terminated the test) are displayed in the screen in tabular format, as well as plotted as a function of the print size (logMAR). The calculated RA, MRS, CPS, and ACC are depicted in Fig. 2b. They are also saved in a file in MS Excel format, or ASCII text (.csv) format. More specifically, the results depict the number of sentences that have been successfully read, as well as the following automatically calculated parameters:

1. the total duration, the initial and ending delay and talk duration (these quantities are described in detail in the next subsection),
2. the reading speed (in wpm),
3. the standard deviation of the reading speed, considering all sentences from the beginning, excluding the current one.
4. RA, MRS, CPS, and ACC (calculated as described before)

### Text size calibration

The *size* of the displayed text is very important for meaningful and accurate visual acuity testing. In theory, text size is defined in terms of physical length of the printed characters. More specifically, for Snellen fraction of 20/20 vision (0.0 logMAR), a printed character should have a height with visual angle of *δφ* = 5 arc minutes [12] when viewed from distance *D* selected for the test, thus, its printed height *H*_0_ should be equal to:

3 $$ {H}_0=D\tan \delta \varphi $$

**Fig. 3:**
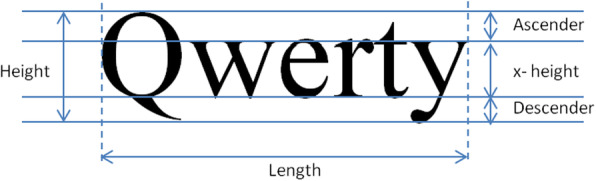
Definition of height and length of text

Τhe height *H*_0_ refers to the main body of the character, called x-height, excluding ascending and descending height, as depicted in Fig. 3 [28, 29]. In the case of near-sight text, the viewing distance *D* is equal to 40 cm, yielding character size *H*_0_ equal to 0.58 mm. For any other logMAR with step of 0.1, the viewing distance is multiplied (for logMAR > 0, equiv. Snellen fraction < 1) or divided (for logMAR < 0, equiv. Snellen fraction > 1) for an appropriate number of times by the factor *r* = 10^0.1^ (= 1.2589), resulting in a text height multiplied or divided an equal number of times by the same factor *r*. Thus, the height of the text for any logMAR at a selected viewing distance *D* is given by:

4 $$ H=D\tan \delta \varphi \cdot {r}^{\log MAR}={H}_0\cdot {r}^{\log MAR} $$

Although it is easy to calculate the necessary font size in points (pt) to achieve the required *printed* size, using the definition of 1 point = 1/72 of an inch (approx. 0.35 mm) [12], it is difficult to guarantee equal size of the height of the *screen-displayed* text. Moreover, the large variety of different pixel resolutions and screen sizes may result in further inaccuracy of the size of the displayed text.

To alleviate this issue, DDART provides an initial text size-calibration feature. A testing sentence appears using the estimated font size for 1.3 logMAR by pushing the “CALIBRATE” button and the user is requested to input its actual length (in cm), as depicted on the screen. Measuring the length of the sentence is equivalent to measuring the x-height of the characters, since a) font resizing maintains the letters’ aspect ratio and b) each one of the three lines of each MNREAD chart sentence contains a standard number of characters (approx. 20, including spaces) [3, 30]. It is considered more accurate as well as convenient for the user to measure the length of the sentence rather than the x-height since it is many times smaller [28, 29].

Considering that the length of the 1st line of the sentence corresponding to 1.3 logMAR should be *L*_0_ = 21 cm (as manually measured from the MNREAD-GR, which is designed to be viewed from distance *D* = 40 cm) the process of size calibration can be described below:

1. The font size is automatically estimated for the 1st sentence (1.3 logMAR) as following: the Snellen fraction for *D* = 40 cm is equal to 20/400, and thus the x-height of a character to appear with an angle of 5 arc min at 20 × *D* is equal to 11.56 mm, which corresponds to font size of 33 pt. (exact value: 32.77 pt) (1 pt. = 1/72 in.).
2. The sentence is displayed with the aforementioned font size and the user measures and inputs the physical length *L* (in cm) of the 1st line.
3. The font rescaling factor is calculated as *L*_0_ / *L*.
4. For each subsequent sentence with a logMAR step of 0.1, the estimated font size is rescaled as described above, before being rendered on screen.

The calibration is required only once for a new display monitor. The accuracy of the x-height of the displayed text for all logMARs, using the aforementioned calibration procedure has been measured experimentally and it is elaborated in the Discussion section. If it is required, DDART can display text of appropriate size for the test to be performed at any viewing distance *D*^′^. More specifically, let *H* be the character x-height for 1.3 logMAR and for *D* = 40 cm, also let *H*^′^ be the x-height for the new viewing distance *D*^′^, as calculated according to Eq. (4). The length of the 1st line of the sentence corresponding to 1.3 logMAR, *L*_0_ = 21 cm is proportionally adjusted: *L*_0_ = (*H*^′^/*H*) ⋅ 21*cm*. Subsequently, the calibration steps 1–4 are repeated.

### Automatic calculation of patient reading times

In DDART, an automatic approach for the measurement of reading duration is used, based on simple signal processing techniques, which is capable of measuring the duration of the talk and pre- and post-talk delays, similarly to the methods used by Radner et al. [13, 14]. More specifically, the patient’s reading is being recorded at sampling frequency *f*_*s*_ = 16 kHz, to create a discrete, signed voltage signal *x*(*n*). After completion of the current chart (sentence), the following algorithm is applied to calculate automatically several timings. First, a threshold *T* is applied to the signal *x*(*n*) to discriminate between noise and useful speech and generates the binary (also called thresholded) signal *b*(*n*) that has only two values: equal to 0 and greater than 0, corresponding to background noise and patient talk, respectively.

5 $$ b(n)=\left\{\begin{array}{c}1,\kern0.75em x(n)\ge T\\ {}0,\mathrm{otherwise}\end{array}\right. $$

Further, morphological processing is applied to signal *b*(*n*), to remove loud but short duration sounds (less than 0.1 s) that may interfere with the accuracy of reading timing. Using the final segmented (binary) signal *b*(*n*), the following time quantities can be easily calculated:

- *total duration* of acquisition, equal to total number of samples × the sampling period (= total number of samples × 1/ *f*_*s*_ = total number of samples × *T*_*s*_)
- *initial delay*: the time between the start of the speech acquisition and the onset of talking, calculated as the number of samples before the first sample *n*_0_, such that *b*(*n*_0_) > 0
- *ending delay*: the time between the end of talking and the end of the speech acquisition, calculated as the number of samples after the last sample *n*_0_, such that *b*(*n*_0_) > 0

The *talk duration* is calculated as the *total duration* minus the *ending delay*. This parameter is used for the calculation of reading speed in Eq. (1).

**Fig. 4:**
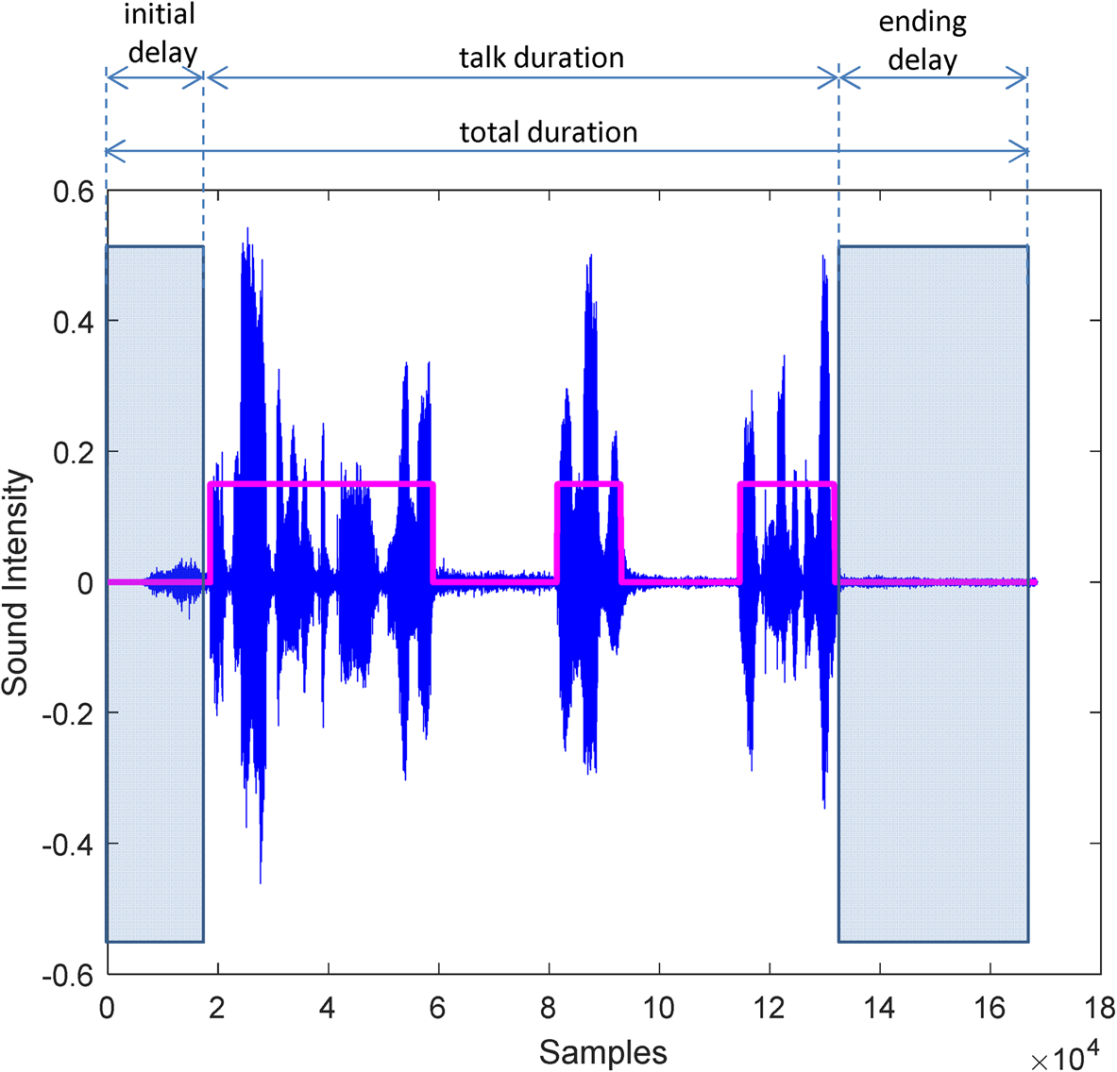
The original signal (blue continuous curve), the segmented patient talk (square – binary signal) and automatic timing, as estimated by the proposed algorithm

Figure 4 shows a typical audio recording of a reading of the 1st sentence (1.3 logMAR). Sound intensity has been normalized to zero-mean, with the sound-segmented signal *b*(*n*), superimposed as a binary (square) signal. Non-zero values of the signal *b*(*n*) indicate the parts of the recording that are considered as ‘talk’ by the algorithm. The initial delay of the patient has been identified, as well as the delay of the examiner to stop the recording. Intermediate pauses have been considered as continuous talk.

### Examination technique

A portable computer with 13.3-in. diagonal display with native resolution of 3840 × 2160 and a pixel density of 331.3 ppi was used to perform the digital reading test. Aforementioned display characteristics are considered as more than sufficient for consistency with the printed MNREAD-GR when not using anti-aliasing displayed fonts. Within this context, the smallest print size that could be displayed with adequate character resolution on the testing screen was between −0.1 and −0.2 logMAR, as it will be discussed in detail in the next section.

One randomly selected eye was included in the study for each study participant. Different versions of character sets in each chart were used in order to avoid the memory effect. In the case of the MNREAD-GR, a uniform environmental lighting of 200 cd/m^2^ was secured. The same environmental lighting conditions were applied for the assessment of DDART; moreover, the computer screen brightness was set to 200 cd/m^2^, as well. Viewing distance was set at 40 cm, participants responded without any spectacle correction first to the MNREAD-GR and then to the DDART; within 15 days, all participants responded again to DDART in a different set of sentences in order to assess its test-retest reliability. All four parameters (RA, MRS, CPS and ACC) were evaluated.

Regarding the examination procedure with MNREAD-GR, each participant masked the sentences using a blank piece of paper and was instructed by an investigator to reveal each sentence and read it aloud, as quickly and accurately as possible, after hearing the words “Ready!… Go!”. At the same time, a second examiner started a stopwatch to record the reading time (in seconds, to the nearest 0.01 s) when the subject fully revealed the sentence and started to read it. The first examiner counted the number of errors (missing words or words read with mistakes) for each sentence. Testing stopped when the print size was too small for the examinee to discriminate the words.

The reading examination with DDART was initiated by clicking the “START” button. Then, the first sentence appeared on the screen and audio recording was initiated. Once reading was completed, the examiner pressed the “STOP” button, to stop the recording. Following every audio recording, the examiner inserted the number of errors made by the patient. Clicking the “NEXT” button proceeded to the next page of DDART that had smaller letters from the previous one by a factor of 10^0.1^ (logMAR step of 0.1) and the same process was repeated. Testing was completed when the “END of PROCESS” button was pressed (patient could not read the sentence) or when all sentences had been read successfully by the patient. The examination was also automatically ended when the patient failed to read the sentence within a specific timeframe, currently set at 30 s.

### Statistical analysis

An a priori power analysis was performed. For an effect size of 0.30 of the RA, 91 participants would be required in total for the study to have a power of 0.8 at the significance level of 0.05. The normality of measured data was evaluated by the Kolmogorov-Smirnov test. Normal distribution data were assessed by Student’s paired samples t-test. Non-parametric data were assessed with Mann–Whitney U test. *P* values less than 0.05 were considered statistically significant. All statistical analyses were performed with the MedCalc version 14.8.1 (MedCalc Software, Mariakerke, Belgium). The same statistical procedure was used to estimate all parameters of the DDART and MNREAD-GR.

The level of agreement between the print and digital version was evaluated by calculation of the intraclass correlation coefficients (ICCs) - two-way mixed, average measures, absolute agreement. Trends in the differences among the two modalities were assessed by Bland-Altman plots. Test-retest reliability of the digital reading test was also evaluated by ICCs (two-way mixed, average measures, absolute agreement) and repeatability Limits of Agreement (LoAs).

## Results

One hundred patients (48 men and 52 women, 50.4 ± 9.8 years) were recruited and responded to both reading tests. Participants were divided according to their distance best-spectacle-corrected visual acuity (BSCVA) in two groups: a) normal vision group (NVG) was populated by 70 participants with BSCVA: 0.02 ± 0.19 logMAR, b) low vision group (LVG) was populated by 30 participants with BSCVA: 0.73 ± 0.35 logMAR. LVG had a wide variety of diagnoses, including age-related macular degeneration (seven), diabetic retinopathy (five), glaucoma (four), optic neuropathy (four), retinitis pigmentosa (three), retinal detachment (two), rod-cone dystrophy (one), myopic degeneration (one), macular hole (one), Stargardt’s disease (one), and congenital cataract (one). Detailed data of both groups are presented in Table 1.

**Table 1:** Demographic and general characteristics

	N	Age (years)	Sex (female / male)	Distance BSCVA (logMAR)	Refractive error (D) (spherical equivalent)
NVG	70	42.03 ± 11.92	38 / 32	0.02 ± 0.19	−0.94 ± 1.19
LVG	30	63.00 ± 16.00	14 / 16	0.73 ± 0.35	−1.02 ± 1.47

*BSCVA* = best-spectacle-corrected visual acuity; *LVG* = low vision group; *N* = number of patients; *NVG* = normal vision group

**Fig. 5:**
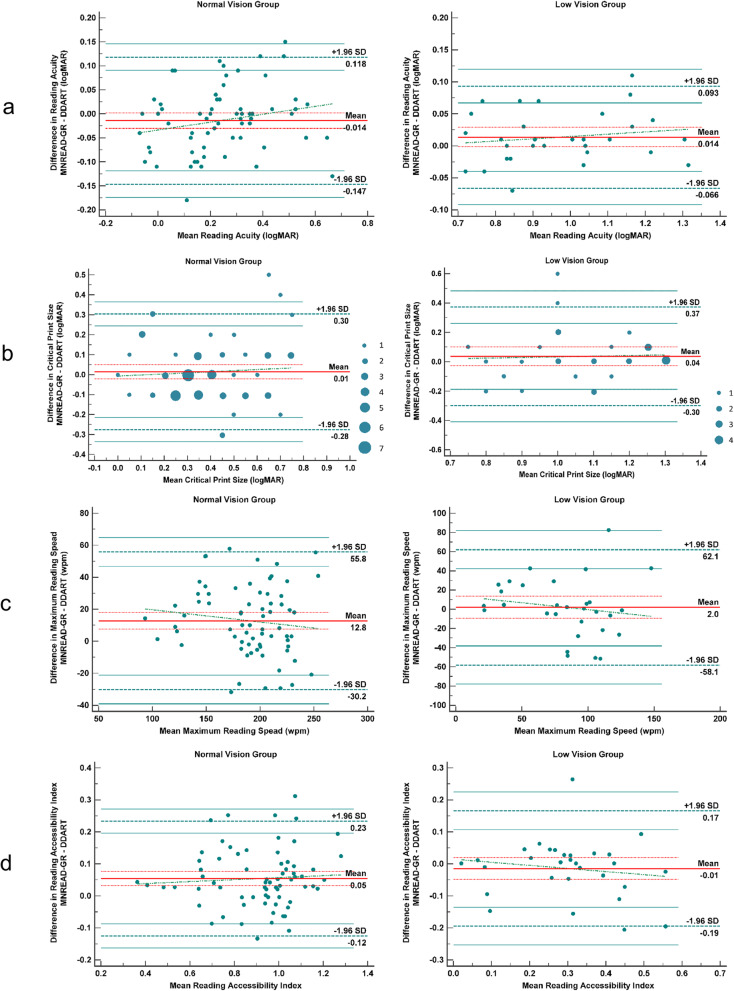
Bland-Altman plots comparing MNREAD-GR and DDART in normal vision group and low vision group (**a**) RA, (**b**) CPS, (**c**) MRS, (**d**) ACC

**Fig. 6:**
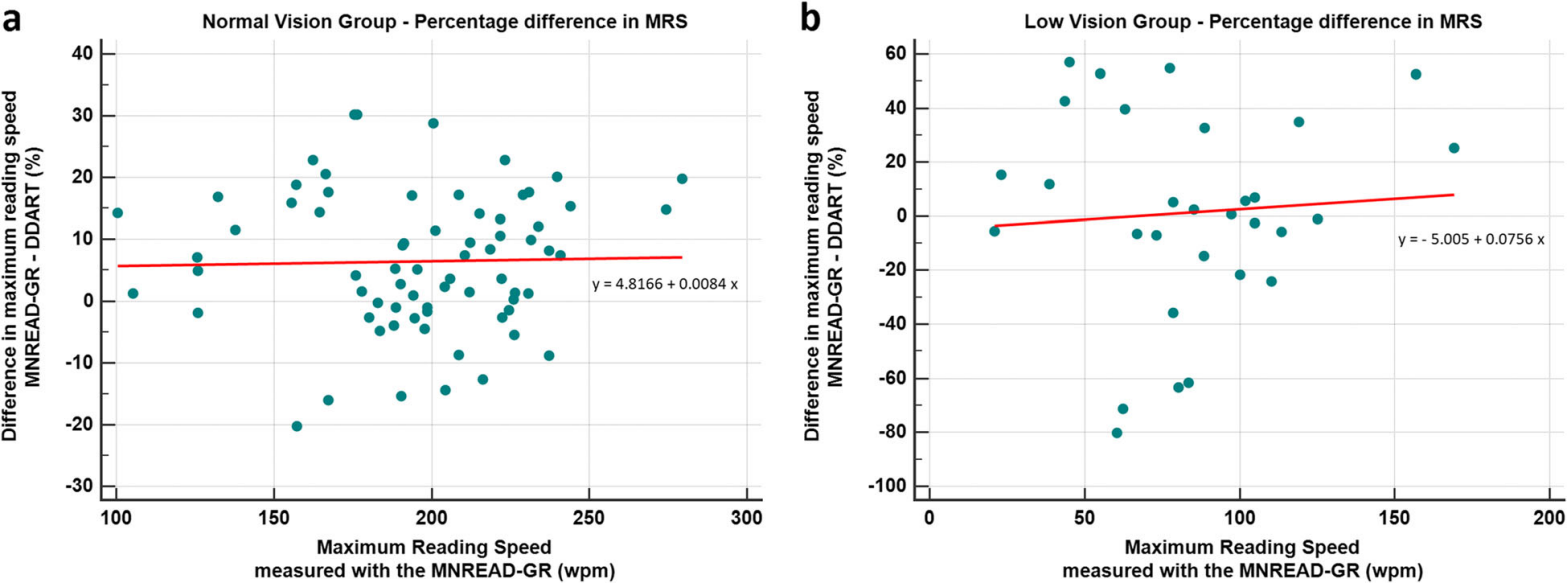
Percentage difference in maximum reading speed (MRS) between MNREAD-GR and DDART as a function of the MNREAD-GR-derived MRS for normal vision group (**a**) and low vision group (**b**)

Comparisons between DDART and MNREAD-GR are presented in Table 2a, b, and c, while Bland Altman plots for all study parameters are presented in Fig. 5 (a-d). Non-significant differences were detected for all studied parameters in LVG participants between DDART and MNRAED-GR (all *p* > 0.05). Regarding NVG participants, non-significant differences were documented in RA (*p* = 0.10; Fig. 5a) and in CPS (*p* = 0.42; Fig. 5b). However, MRS was significantly faster in MNREAD-GR than in DDART (*p* <  0.001; Fig. 5c). Figure 6 analyses the correlation between the value of the MNREAD-GR MRS and the percentage MRS difference between MNREAD-GR and DDART for NVG and LVG patients, respectively. More specifically, the percentage MRS difference for the NVG patients is almost independent from the MNREAD-GR MRS (ranging between 5.7% at 100 wpm and 6.9% at 250 wpm). On the other hand, for the LVG patients, the percentage MRS difference increases with the MNREAD-GR MRS values from 2.6% at 100 wpm to 13.9% at 250 wpm. Finally, significant differences were also detected in ACC between the two reading tests (*p* <  0.001; Fig. 5d).

**Table 2:** Comparison of reading parameters

**Normal Vision Group**				
**Reading parameter**	**Mean ± SD [95% CI]**		**Difference ± SD [95% CI]**	**p**
	**MNREAD-GR**	**DDART**		
RA (logMAR)	0.23 ± 0.19 [0.18, 0.27]	0.24 ± 0.17 [0.20, 0.28]	−0.01 ± 0.07 [−0.03, 0.002]	0.10
MRS (wpm)	196.36 ± 36.09 [187.75, 204.96]	183.56 ± 38.71 [174.33, 192.79]	12.8 ± 21.93 [7.57, 18.03]	< 0.001*
CPS (logMAR)	0.40 ± 0.21 [0.35, 0.45]	0.385 ± 0.20 [0.34, 0.43]	0.015 ± 0.15 [−0.02, 0.05]	0.42
ACC	0.94 ± 0.20 [0.89, 0.99]	0.885 ± 0.20 [0.84, 0.93]	0.055 ± 0.10 [0.03, 0.08]	< 0.001*
**Low Vision Group**				
**Reading parameter**	**Mean ± SD [95% CI]**		**Difference ± SD [95% CI]**	**p**
	**MNREAD-GR**	**DDART**		
RA (logMAR)	0.98 ± 0.18 [0.91, 1.05]	0.97 ± 0.18 [0.90, 1.03]	0.01 ± 0.04 [−0.002, 0.03]	0.08
MRS (wpm)	83.88 ± 34.47 [71.01, 96.75]	81.89 ± 38.87 [67.38, 96.4]	1.99 ± 30.67 [−9.46, 13.44]	0.73
CPS (logMAR)	1.09 ± 0.19 [1.02, 1.16]	1.055 ± 0.18 [0.99, 1.12]	0.035 ± 0.17 [−0.03, 0.10]	0.25
ACC	0.295 ± 0.14 [0.17, 0.42]	0.31 ± 0.15 [0.18, 0.43]	−0.015 ± 0.09 [−0.05, 0.02]	0.40
**All study participants**				
**Reading parameter**	**Mean ± SD [95% CI]**		**Difference ± SD [95% CI]**	**p**
	**MNREAD-GR**	**DDART**		
RA (logMAR)	0.45 ± 0.39 [0.38, 0.53]	0.46 ± 0.37 [0.39, 0.53]	−0.006 ± 0.06 [−0.02, 0.01]	0.34
MRS (wpm)	162.61 ± 62.77 [150.16, 175.07]	153.06 ± 60.66 [141.02, 165.09]	9.55 ± 25.21 [−4.55, 14.56]	< 0.001*
CPS (logMAR)	0.61 ± 0.38 [0.53, 0.68]	0.59 ± 0.37 [0.51, 0.66]	0.02 ± 0.15 [−0.01, 0.05]	0.18
ACC	0.74 ± 0.35 [0.68, 0.81]	0.71 ± 0.32 [0.65, 0.78]	0.03 ± 0.10 [0.01, 0.05]	< 0.001*

*p < 0.05

*ACC* = reading accessibility index; *CI* = confidence interval; *CPS* = critical print size; *DDART* = Democritus Digital Acuity Reading Test; *MNREAD-GR* = Greek version of the MNREAD acuity chart; *MRS* = maximum reading speed; *RA* = reading acuity; *SD* = standard deviation; *wpm* = words per minute

ICCs for all parameters between the two charts are presented in Table 3. NVG participants demonstrated high correlation for all parameters (RA, MRS, CPS, and ACC; ICCs: 0.854 to 0.963), while LVG participants presented high correlation for RA and ACC (ICCs: 0.986 and 0.894 respectively), and average correlation for MRS and CPS. (ICCs: 0.794 and 0.723 respectively). Test-retest reliability ICCs and repeatability LoAs for DDART are presented in Table 4. All parameters demonstrated excellent reliability (ICCs: 0.903 to 0.956).

**Table 3:** Intraclass correlation coefficients for study participants

Parameter	NVG		LVG		Total	
	ICC	95% CI	ICC	95% CI	ICC	95% CI
RA	0.963	[0.940, 0.977]	0.986	[0.969, 0.993]	0.993	[0.990, 0.996]
MRS	0.879	[0.718, 0.939]	0.794	[0.565, 0.902]	0.951	[0.917, 0.970]
CPS	0.854	[0.766, 0.910]	0.723	[0.425, 0.867]	0.955	[0.933, 0.970]
ACC	0.928	[0.819, 0.965]	0.894	[0.779, 0.950]	0.977	[0.962, 0.985]

[ICCs: two-way mixed, average measures, absolute agreement]

*ACC* = reading accessibility index; *CI* = confidence interval; *CPS* = critical print size; *ICC* = intraclass correlation coefficient; *LVG* = low vision group; *MRS* = maximum reading speed; *NVG* = normal vision group; *RA* = reading acuity

**Table 4:** Test-retest intraclass correlation coefficients – repeatability limits of agreement

Parameter	NVG			LVG			Total		
	ICC	95% CI	LoA	ICC	95% CI	LoA	ICC	95% CI	LoA
RA	0.914	[0.901, 0.928]	[−0.163, 0.174]	0.928	[0.905, 0.947]	[−0.134, 0.196]	0.922	[0.897, 0.941]	[−0.151, 0.165]
MRS	0.956	[0.944, 0.971]	[−91.565, 43.947]	0.943	[0.914, 0.968]	[−87.599, 39.762]	0.944	[0.913, 0.972]	[−89.548, 41.857]
CPS	0.909	[0.889, 0.931]	[−0.394, 0.234]	0.903	[0.881, 0.928]	[−0.318, 0.188]	0.912	[0.887, 0.931]	[−0.349, 0.199]
ACC	0.927	[0.898, 0.949]	[−0.455, 0.380]	0.926	[0.885, 0.961]	[−0.258, 0.146]	0.928	[0.911, 0.953]	[−0.398, 0.311]

[ICCs: two-way mixed, average measures, absolute agreement]

*ACC* = reading accessibility index; *CI* = confidence interval; *CPS* = critical print size; *ICC* = intraclass correlation coefficient; *LVG* = low vision group; *MRS* = maximum reading speed; *NVG* = normal vision group; *RA* = reading acuity

## Discussion

### Validation outcomes

In contrast to the standardized modern log-scaled reading charts [3, 7], the still prevalent in clinical practice not standardized near vision Jaeger test does not represent a comparable standard for the evaluation of reading capacity. Additionally, it only estimates the patients’ reading acuity without considering the patients’ fluency. As a result, the assessment of reading capacity is usually incomplete. On the other hand, the standardized modern reading charts are gradually gathering clinical acceptance. For instance, the MNREAD chart is a tool capable of evaluating more effectively the reading capacity, since it uses not only the reading acuity (RA), but also other parameters: critical print size (CPS), maximum reading speed (MRS), reading accessibility index (ACC). However, these parameters are difficult to be calculated during the test, when the printed reading test is used. On the other hand, a digital reading chart that is able to automatically calculate these parameters at the end of the test would be very useful both for the examiner and the patient.

The proposed DDART belongs to the family of digital visual acuity tests, like the MNREAD [3] and RADNER test [13–15]. It contains the fundamental layout and linguistic principles of the MNREAD-GR with a series of digital user-friendly enhancements; among them, a) screen calibration for ensuring the correct size of the text in different computer screens, b) automatic recording and timing of the patient’s response, and c) real-time calculation and display of all parameters.

Regarding the validation process, non-significant differences in all parameters for LVG participants and in RA and CPS for the NVG participants were observed. These findings are in accordance with Calabrèse at al [16]. who reported non-significant differences as well. On the other hand, Kingsnorth et al. [15] compared the conventional and an iPad app of the RADNER Reading Chart and found that CPS was significantly lower in the digital version. This may be attributed to the fact that MNREAD uses 60 characters including spaces with different number of words and difficulty, whereas the RADNER charts use more equivalent sentences, and therefore identify differences in reading parameters more accurately.

In our study, MRS and ACC were significantly higher in MNREAD-GR in NVG participants. However, such differences in MRS and ACC were encountered in previous reports, as well. Calabrèse et al. [16] reported an average difference of 16 wpm (MRS) and 0.09 (ACC) compared to 12.8 wpm (MRS) and 0.055 (ACC) in our case. It should be mentioned that both in our study and in the study of Calabrèse et al. [16], there is a positive correlation between the reading speed of the patient and the difference in MRS between a digital and conventional printed acuity test. According to Xu & Bradley [20], this finding is attributed to the underestimation of reading time resulting in the overestimation of reading speed when using a stopwatch against a computer-based timing method. When a voice detection system is implemented in the examination procedure like in the Salzburg reading desk, differences in MRS become non-significant [21]. Finally, it is worth noting that the mean RA of our NVG and LVG patients for both reading tests was lower than the respective RA values found by Calabrèse [16], as they are analyzed in Table 5. This difference may be attributed to the fact that our patients were examined without any spectacle correction, while Calabrèse’s participants were examined with their best near correction.

**Table 5:** Comparison of reading parameters between digital and printed optotype in the proposed method and in the study of Calabrèse et al. [16]

	Patient group	This study Mean ± SD [95% CI]		Calabrèse et al. [16] Mean ± SD [95% CI]	
		MNREAD-GR	DDART	Printed	Digital
**RA** (logMAR)	NVG	0.23 ± 0.19 [0.18, 0.27]	0.24 ± 0.17 [0.20, 0.28]	−0.13 ± 0.10 [−0.15, −0.12]	−0.14 ± 0.20 [−0.17, −0.11]
	LVG	0.98 ± 0.18 [0.91, 1.05]	0.97 ± 0.18 [0.90, 1.03]	0.77 ± 0.40 [0.65, 0.89]	0.74 ± 0.47 [0.60, 0.88]
**MRS** (wpm)	NVG	196.36 ± 36.09 [187.75, 204.96]	183.56 ± 38.71 [174.33, 192.79]	182 ± 39.3 [174, 186]	166 ± 39.32 [158, 170]
	LVG	83.88 ± 34.47 [71.01, 96.75]	81.89 ± 38.87 [67.38, 96.4]	85 ± 48.5 [71, 100]	83 ± 60.2 [68, 104]
**CPS** (logMAR)	NVG	0.40 ± 0.21 [0.35, 0.45]	0.385 ± 0.20 [0.34, 0.43]	0.09 ± 0.07 [0.09, 0.11]	0.06 ± 0.33 [0.01, 0.11]
	LVG	1.09 ± 0.19 [1.02, 1.16]	1.055 ± 0.18 [0.99, 1.12]	1.02 ± 0.75 [0.9, 1.13]	1.00 ± 0.98 [0.85, 1.15]
**ACC**	NVG	0.94 ± 0.20 [0.89, 0.99]	0.885 ± 0.20 [0.84, 0.93]	0.92 ± 0.16 [0.89, 0.94]	0.83 ± 0.26 [0.79, 0.87]
	LVG	0.295 ± 0.14 [0.17, 0.42]	0.31 ± 0.15 [0.18, 0.43]	0.36 ± 0.22 [0.29, 0.42]	0.37 ± 0.28 [0.28, 0.45]

*ACC* = reading accessibility index; *CPS* = critical print size; *DDART* = Democritus Digital Acuity Reading Test; *LVG* = low vision group; *MNREAD-GR* = Greek version of the MNREAD acuity chart; *MRS* = maximum reading speed; *NVG* = normal vision group; *RA* = reading acuity; *wpm* = words per minute

The non-significant differences in RA between the MNREAD-GR and the DDART are associated with: a) the adequate validity of DDART both in normal and low vision patients, b) its capability to display even the smallest print size sentences, in specific video screens, c) the fact that the exact same lighting conditions were secured during testing with both charts, and, d) the fact that the exact same viewing distance was used with both tests. All aforementioned conditions should be addressed in order to ensure reliable and replicable results. For example, the significant better scores in RA that were detected by Tofigh et al. [31], in their report of the Eye Handbook application, were attributed to the increased contrast of the smartphone’s screen compared to the printed reading test.

### Validation of displayed text size and minimal technical specifications of the screen for the near-vision DDART

The actual size of the displayed text and the required specifications of the display unit are of critical importance for a reliable vision test. In digital displays, the vertical pixel density, measured in pixels per inch (ppi) or equivalently pixels per centimeter (ppcm), defines the smallest displayable character height. It has to be emphasized that for a digital display to be utilized for a near-vision test, it has to be able to display small prints legibly. In case of non-smoothed text (where no anti-aliasing technique has been applied), it is assumed that at least 5 pixels along the x-height (without ascenders and descenders – see Fig. 3) are necessary for character display. For 0.0 logMAR, one pixel should span a visual angle of 1 arc minute [32], and thus for any logMAR value the theoretical x-height *H* of text is provided according to Eq. (4) and the required vertical pixel density (number of pixels per inch -ppi- and per centimeter- *ppcm*) to display correctly aliased text is given by:

6 $$ ppi=25.4\cdot \frac{5}{H}, ppcm=\frac{5}{H} $$

**Fig. 7:**
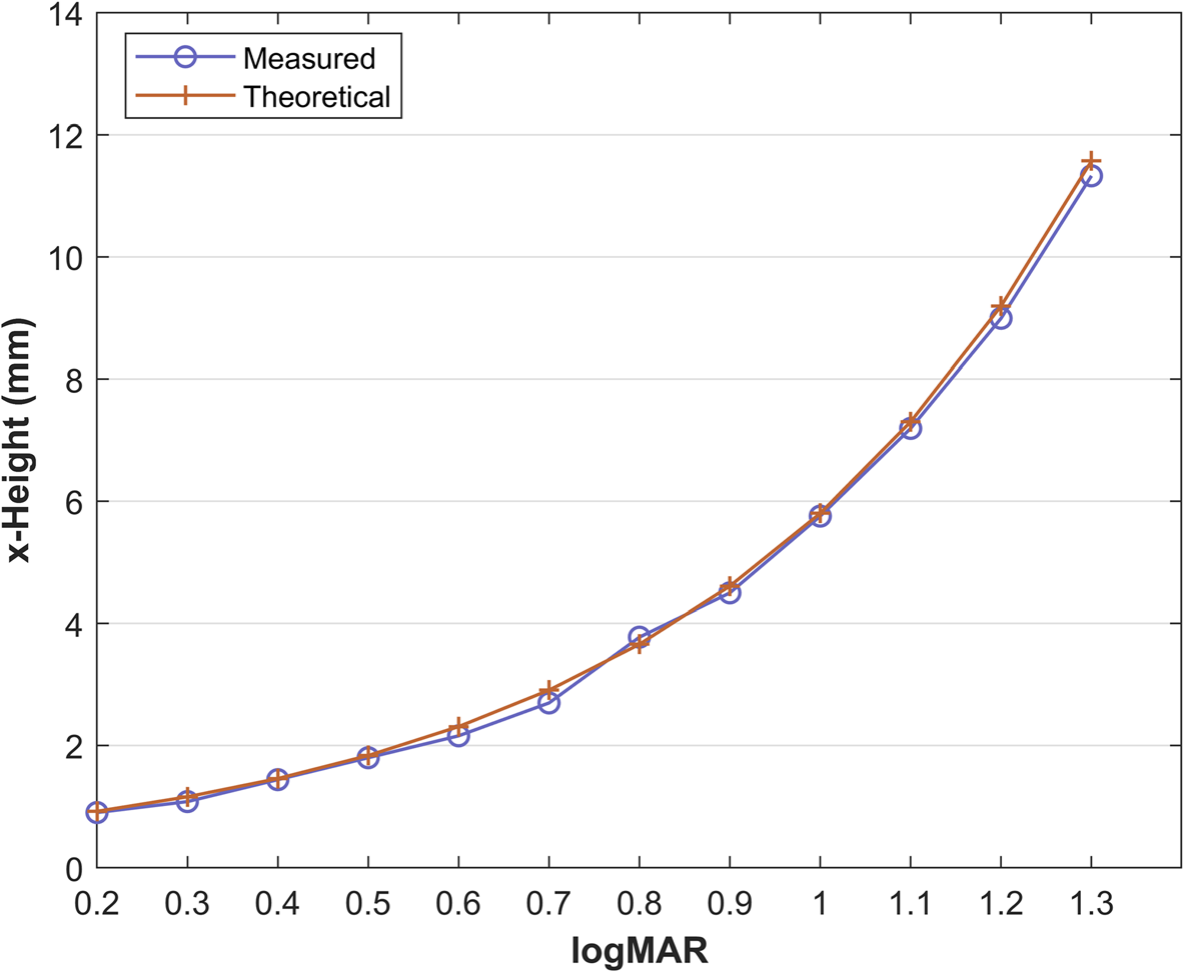
Percentage error of the actual size of the aliased text displayed using DDART compared with the theoretical size

In order to validate the displayed x-height, an experiment was carried out using a number of available computer screens. Details are presented for a full high definition (FHD) laptop screen (1920 × 1080) with a 15.6 in. diagonal that achieves vertical *ppi* = 141.26. The testing screen was calibrated using the aforementioned process and the set of characters “eg” were printed at the estimated font size, setting the “FontSmoothing” property to “off”. The text rendered on the computer screen was photographed using a Canon EOS 250D DSLR camera (exposure and focus was set to manual), so that the individual screen pixels are clearly visible. The x-height was measured in pixels from the photographs and then converted to actual length (mm) using the screen’s ppi. The theoretically expected x-height was also calculated using Eq. (4). Table 6 shows the aforementioned quantities for 1.3 logMAR to 0.2 logMAR (the specific testing screen is not capable to legibly display aliased characters smaller than 0.2 logMAR at 40 cm, as it will be discussed). The cropped photos of logMAR less than or equal to 0.7 are also provided. It can be visually verified that the 0.2 logMAR text is marginally readable, as theoretically expected from Table 7. Selecting a screen with smaller physical size and the same or better pixel resolution, will enable rendering smaller logMAR for the 40 cm viewing distance. The percentage error between the actual and the theoretical x-height is shown in Table 6, as well as plotted in Fig. 7 for different logMARs. It can be observed that the mean error of the aliased text size displayed by the proposed DDART is 3.18% and the maximum error is 7.21% (for 0.7 logMAR). Therefore, the proposed software can be calibrated for different screens to render the testing text with accurate actual size. The calibration is a very simple and quick process that requires no expert knowledge and it is necessary only once for a new display monitor.

**Table 6:** The required aliased character x-height and the necessary vertical pixel density of the displaying screen for performing the near-vision test at viewing distance *D* = 40cm

Actual photos of displayed text and x-height in pixels	logMAR	Snellen	Character x-height (mm)			Minimum required screen pixels/inch (ppi)
			Theoretical	Measured	Error %	
63 pixels	1.3	6/120	11.56	11.33	2.11	10.99
50 pixels	1.2	6/95	9.18	9.99	2.19	13.83
40 pixels	1.1	6/76	7.30	7.19	1.50	17.40
32 pixels	1.0	6/60	5.80	5.75	0.79	21.90
25 pixels	0.9	6/48	4.61	4.50	2.43	27.54
21 pixels	0.8	6/38	3.66	3.78	3.19	34.66
	0.7	6/30	2.91	2.70	7.21	43.64
	0.6	6/24	2.3	2.16	6.55	54.98
	0.5	6/20	1.84	1.80	1.96	69.13
	0.4	6/15	1.46	1.44	1.26	86.98
	0.3	6/12	1.16	1.08	6.77	109.48
	0.2	6/10	0.92	0.90	2.19	138.37
Too small character for useful display with the specific screen	0.1	6/8	0.73	N/A	N/A	175.17
	0.0	6/6	0.58	N/A	N/A	218.97
	-0.1	6/4.8	0.46	N/A	N/A	273.71
	-0.2	6/3.8	0.37	N/A	N/A	346.19
	-0.3	6/3	0.29	N/A	N/A	437.93

*ppi* pixels per inch, *N/A* not applicable

**Table 7:** Technical specifications of prevalent computer screens and calculation of the minimum and maximum displayable aliased text size (logMAR) for a viewing distance of 40 cm

Screen specifications						logMAR at 40 cm	
Resolution	Diagonal (inch)	pixels	size (cm)	pixel size (mm)	ppi	Min	Max
FHD	15.6	1920 × 1080	34.54 × 19.42	0.18	141.26	0.2	> 1.3
FHD	14	1920 × 1080	30.99 × 17.43	0.16	157.38	0.2	> 1.3
4 K	15.6	3840 × 2160	34.54 × 19.42	0.09	282.4	−0.1	> 1.3
4 K	14	3840 × 2160	30.99 × 17.43	0.081	314.7	< −0.1	> 1.3
4 K	13.3	3840 × 2160	29.44 × 16.56	0.08	331.3	< −0.1	> 1.3
2.5 K	10	2560 × 1440	22.14 × 12.45	0.09	293.78	−0.1	1.3

*FHD* = full high definition; *ppi* = pixels per inch

A brief analysis of the specifications of some popular available computer displays can determine their applicability for near-vision DDART with aliased fonts. Although a large variety of screen resolutions and sizes can be found in commercial computer hardware, the family of laptops/ultrabooks is dominated by FHD (1920 × 1080 pixels) and 4 K resolution screens (3840 × 2160 pixels), as well as an intermediate 2.5 K resolution (2560 × 1440 pixels), each family with a variety of different diagonal screen lengths. Table 7 provides relevant details for FHD, 4 K and 2.5 K screen resolutions for different lengths of screen diagonal [33, 34]. More specifically, the value of ppi and the minimum and maximum displayable logMAR at viewing distance of 40 cm are provided.

In principle, the resolution of the screen (in pixels) and its physical size (in inches) can be obtained by the proposed DDART; thus, the minimum allowable print size could be calculated. However, the obtained values may vary between different versions of the programming environment (MATLAB) and different operating systems (Windows, MacOS, Linux). Therefore, it is safest to manually calculate the screen’s ppi before using the application for the first time with the specific screen.

A number of netbooks and tablets with 4 K digital displays (3840 × 2160 pixels) with appropriate diagonal length are able to render aliased text up to −0.1 logMAR at 40 cm, since they achieve vertical *ppi* > 273, whereas their horizontal length is sufficient for 1.3 logMAR (the horizontal length of the display screen has to exceed 21 cm for 1.3 logMAR). If the family of the latest smart phones is considered, then the combination of small screen sizes (approximately 5 to 6 in.) with the very high available resolutions, allow for very high values of ppi, usually higher that 500 ppi, thus enabling the display of text at −0.3 or even −0.4 logMAR. However, the small physical dimensions of the screen prevent the display of text larger than 1.0 logMAR. If a specific display device cannot support rendering of very small (or very large) text sizes (to satisfy logMAR for 40 cm viewing distance), then the viewing distance for the smaller (or larger prints) may be modified [20].

## Conclusions

The development and validation of a contemporary digital near-vision test for Greek-speaking patients (DDART), capable of audio recording and real-time calculation of all reading parameters, has been reported. The proposed DDART presented comparable validity and repeatability to MNREAD-GR, suggesting that it can be used both in normal and low vision Greek patients. Differences in MRS and ACC are considered to derive from suboptimal measurement of patient’s response times with a stopwatch when using the MNREAD-GR.

DDART is a user-friendly reading tool that can be installed in any Windows-based computer provided that its screen has adequate size, resolution and pixel density to display correctly all set of sentences. Moreover, it is easily upgradable when new features or parameters are introduced indicating that it can be used in clinical and research settings.

## Data Availability

De-identified data are available in print form for 1 year following the conclusion of the study.

## Abbreviations

ACC: Reading Accessibility Index
BSCVA: Best-Spectacle-Corrected Visual Acuity
CPS: Critical Print Size
DDART: Democritus Digital Acuity Reading Test
FHD: Full High Definition
ICCs: Intraclass Correlation Coefficients
LoAs: Limits of Agreement
LVG: Low Vision Group
MNREAD-GR: Greek version of the MNREAD acuity chart
MRS: Maximum Reading Speed
NVG: Normal Vision Group
ppcm: Pixels per centimeter
ppi: Pixels per inch
pt.: Points
RA: Reading Acuity
SD: Standard Deviation
wpm: Word per minute
